# The effects of Traditional Chinese Medicine on cardiac function after percutaneous coronary intervention: a meta-analysis and systematic review

**DOI:** 10.3389/fcvm.2026.1619928

**Published:** 2026-02-18

**Authors:** Mengchen Wang, Shuai Fan, Libo Xia, Zixu Wang, Jixiang Ren

**Affiliations:** 1 College of Traditional Chinese Medicine, Changchun University of Chinese Medicine, Changchun, Jilin, China; 2 Affiliated Hospital to Changchun University of Chinese Medicine, Changchun, Jilin, China

**Keywords:** cardiovascular disease, coronary artery disease, integrated Chinese and western medicine therapy, meta-analysis, PCI-percutaneous coronary intervention

## Abstract

**Objective:**

This systematic review aimed to synthesize and summarize current evidence regarding the effects of Traditional Chinese Medicine (TCM) on cardiac function in patients with coronary heart disease (CHD) following percutaneous coronary intervention (PCI).

**Methods:**

A comprehensive search was conducted in PubMed, Embase, the Cochrane Library, China National Knowledge Infrastructure (CNKI), Wanfang Database, the Chinese Biomedical Literature Database (CBM), and the Chinese Scientific and Technological Journal Database (VIP). Relevant conference papers were also manually screened. Studies evaluating the clinical efficacy of TCM combined with conventional Western medicine for CHD after PCI were selected based on predefined inclusion and exclusion criteria. The methodological quality of the included studies was assessed using the RoB 2. Data extraction was performed independently, and Stata software was used for meta-analysis.

**Results:**

12 studies involving a total of 6,383 patients were included. Compared with conventional Western medicine alone, integrated TCM and Western therapy significantly reduced the incidence of in-stent restenosis (ISR) and non-fatal myocardial infarction after PCI (*p* < 0.05). However, no significant differences were observed in cardiac mortality or the incidence of coronary artery bypass grafting (CABG) between the two treatment approaches (*p* > 0.05).

**Conclusion:**

The combination of TCM with conventional Western therapy may reduce ISR and non-fatal myocardial infarction in CHD patients after PCI compared with Western medicine alone, suggesting potential benefits for improving post-PCI clinical outcomes.

## Introduction

Coronary heart disease (CHD) remains a major global health challenge, affecting millions of individuals worldwide. The prevalence of cases of CHD was estimated to be about 197 million in 2019 worldwide (1). According to World Health Organization (WHO), CVD also accounted for the largest proportion of all deaths in China (2). It is primarily driven by coronary atherosclerosis and arterial spasm, which lead to luminal stenosis and, in severe cases, complete occlusion (3). These pathological changes result in myocardial ischemia, hypoxia, and ultimately necrosis, with chest pain serving as the predominant clinical manifestation. In China, both the morbidity and mortality associated with CHD continue to rise, making it the leading cause of death among the population (4). Furthermore, CHD imposes a substantial burden on healthcare systems and exerts significant economic pressure on society.

Current treatment strategies for CHD include pharmacotherapy, percutaneous coronary intervention (PCI), and coronary artery bypass grafting (CABG). Among these, PCI is widely utilized to alleviate vascular stenosis and restore myocardial perfusion (5). It is favored by patients because of its advantages, including accurate lesion assessment, effective clinical outcomes, minimal invasiveness, reduced procedural risks, and rapid postoperative recovery (5–7). However, the growing use of PCI has also highlighted several limitations. These include its inability to fully restore perfusion to damaged myocardium, the risk of secondary vascular endothelial injury, abnormalities in myocardial microcirculation, and insufficient reperfusion at the cellular level following the procedure (4, 8, 9). As a result, postoperative cardiac function recovery may be suboptimal in some patients.

Traditional Chinese medicine (TCM), with a history spanning several millennia, has attracted increasing attention for its potential in treating a wide range of diseases, including cardiovascular disorders. TCM offers unique advantages in preventing the recurrence of CHD and non-fatal myocardial infarction following PCI. Its holistic therapeutic philosophy emphasizes patient-centered care and the harmonization of physiological and psychological states. Various TCM modalities—including herbal medicine, acupuncture, moxibustion, and Tui Na massage—have been employed to support postoperative recovery in patients with CHD by enhancing myocardial perfusion, reducing oxidative stress, and mitigating inflammatory responses (10–13).

In recent years, increasing attention has been directed toward the integration of TCM and Western medicine as a potential strategy for improving the prognosis of patients with CHD. This integrative approach seeks to harness the complementary strengths of both medical systems—combining the holistic, individualized principles of TCM with the evidence-based, targeted interventions of Western medicine—to optimize patient care. Several studies have reported encouraging outcomes when TCM is used alongside conventional therapies, such as PCI, suggesting synergistic effects that may enhance overall treatment efficacy (14). For example, certain TCM herbal formulations have been shown to improve myocardial perfusion, reduce inflammation, and modulate oxidative stress, thereby potentially augmenting the benefits of PCI in restoring blood flow to ischemic myocardium and supporting postoperative cardiac function recovery. In addition, TCM interventions such as acupuncture and moxibustion have demonstrated potential in managing postoperative pain, reducing psychological stress, and improving quality of life [as evidenced by improvements in Seattle Angina Questionnaire (SAQ) and SF-36 scores] among CHD patients undergoing PCI (15). Despite these promising findings, the current evidence base regarding the efficacy and safety of TCM in the postoperative management of CHD remains limited and inconclusive.

Given the rising prevalence of CHD and the limitations of current treatment strategies, it is essential to explore alternative or complementary therapies that may enhance patient outcomes (16, 17). The objective of this systematic review is to synthesize and evaluate the existing literature on the effects of TCM on cardiac function following PCI. By providing a comprehensive and evidence-based assessment, this review aims to offer a scientific foundation and theoretical framework for the clinical application of TCM in CHD management. Ultimately, by integrating the available evidence, we seek to support the development of more effective and personalized treatment strategies for CHD patients and help reduce the global burden of this debilitating disease.

## Materials and methods

This systematic review was conducted, and the results were reported in accordance with the Preferred Reporting Items for Systematic Reviews and Meta-Analyses (PRISMA) guidelines (10). Ethical approval and informed consent were not required, as all data were derived from previously published studies. Two researchers independently performed the literature search, study selection, data extraction, and quality assessment. Any discrepancies were resolved through discussion until consensus was achieved.

### Search strategy

A comprehensive literature search was conducted using electronic databases, including PubMed, Embase, the Cochrane Library, CNKI, and the Wanfang Database. Both controlled vocabulary and free-text terms were applied, incorporating keywords such as “coronary heart disease,” “Chinese medicine,” “stenting,” and “randomized controlled trial.” The search covered all available studies from database inception to March 31, 2025. The detailed search strategies for each database are provided in Supplementary Table S1.

### Inclusion criteria and exclusion criteria

To be eligible for inclusion in this systematic review, studies were required to meet the following criteria:(1) randomized controlled trials (RCTs), with no restrictions on blinding; (2) participants diagnosed with CHD confirmed by coronary angiography and treated with PCI; (3) intervention measures in which the treatment group received TCM (including herbal decoctions, injections, or proprietary formulations—either compound preparations, single agents, or extracts) in combination with conventional Western medicine, while the control group received conventional Western medicine alone; (4) no restrictions on gender, age, occupation, nationality, education level, or ethnicity, and comparable baseline characteristics between groups; (5) reported outcome measures focusing primarily on adverse cardiovascular events, including ISR, non-fatal myocardial infarction, cardiogenic death, and CABG.

The exclusion criteria were as follows: (1) non-randomized controlled trials, animal studies, or *in vitro* experiments; (2) studies lacking clear or extractable statistical data; (3) participants with severe malignant arrhythmias or severe dysfunction of major organs such as the lungs, liver, or kidneys; (4) case reports, commentaries, expert opinions, and narrative reviews.

### Data extraction

Literature screening and data extraction were performed independently by two reviewers and subsequently cross-checked. Any discrepancies were resolved through discussion until consensus was reached. During the screening process, titles and abstracts were first reviewed to exclude clearly irrelevant studies, after which the full texts of potentially eligible articles were assessed to determine inclusion or exclusion. Relevant data were extracted into standardized Excel spreadsheets, including the first author's surname, year of publication, country, study design, participant demographics, and outcome measures such as ISR, nonfatal myocardial infarction, cardiogenic death, and CABG. When data of interest were not reported in the published articles, the corresponding authors of the original studies were contacted by email to request the missing information.

### Risk of bias assessment

Since all included studies were randomized controlled trials (RCTs), the Cochrane Risk of Bias Tool 2.0 (RoB 2) was used to systematically assess the methodological quality, in accordance with the Cochrane Handbook for Systematic Reviews of Interventions. This tool evaluates five core domains of bias that directly affect the validity of RCTs. Each domain was rated as “Low risk of bias,” “High risk of bias,” or “Some concerns,” based on the explicit criteria outlined in the RoB 2 manual. Two researchers independently completed the assessment, and any discrepancies were resolved through discussion until consensus was reached.

### Statistical analyses

The degree of heterogeneity among the included studies was assessed using chi-square statistics and further quantified by the I² statistic. An I² value of 0% indicated no observed heterogeneity, whereas values greater than 50% suggested substantial heterogeneity. The relative risk reported in each study was standardized and pooled using a random-effects model. In addition, a sensitivity analysis was performed to determine whether any single study had a significant influence on the overall results using the leave-one-out approach. Publication bias for meta-analyses that included 10 or more studies was examined by assessing the symmetry of funnel plots and conducting Egger's test. When funnel plot asymmetry was detected, hypothetical unpublished negative studies were imputed to evaluate whether publication bias materially affected the effect estimates. A two-sided *p*-value < 0.05 was considered statistically significant for all analyses. Data from randomized controlled trials that met the inclusion criteria were analyzed using Stata version 17 (StataCorp, College Station, TX, USA).

## Results

### Search results and study selection

**Figure 1:**
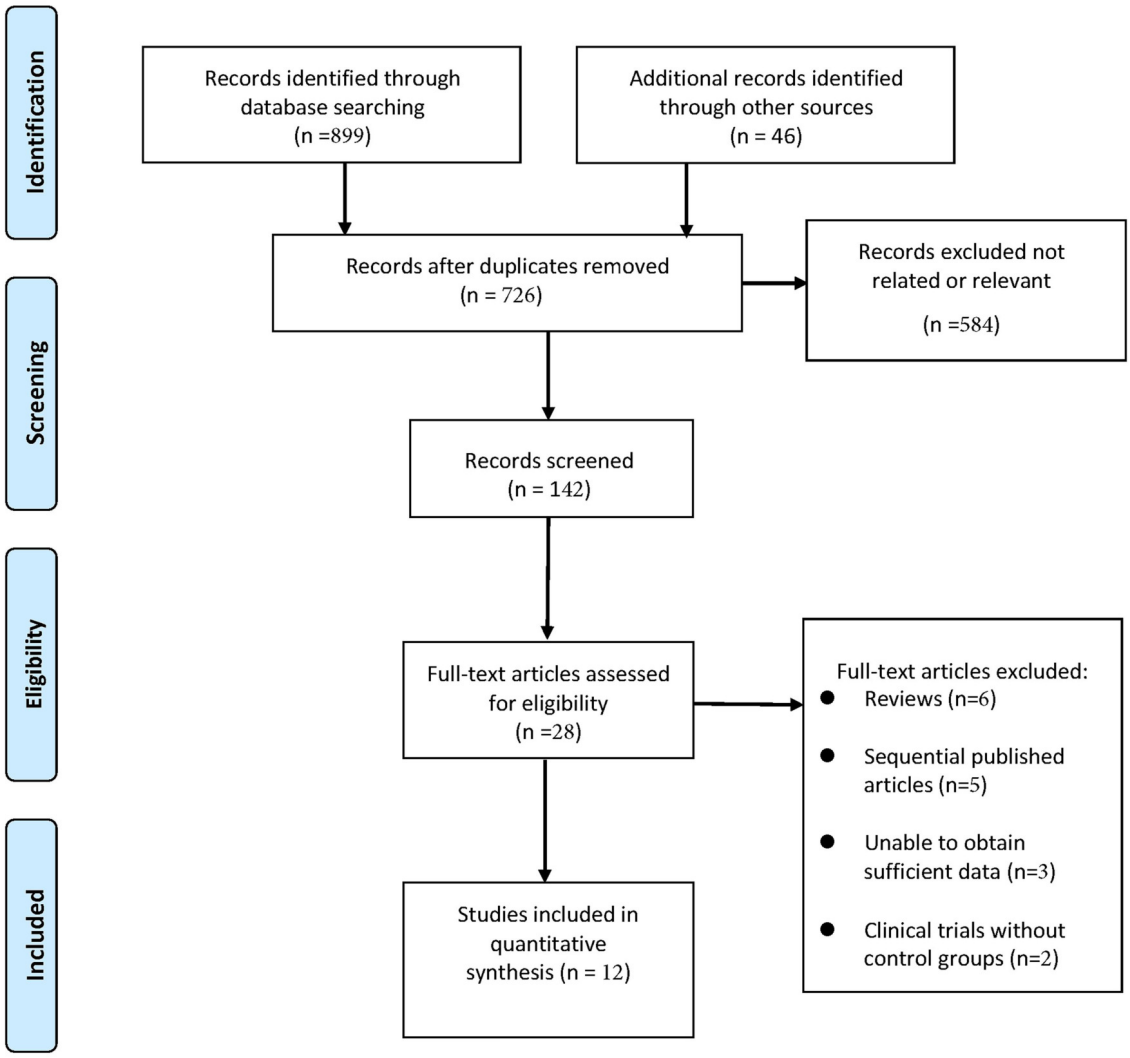
Selection process of included studies.

An initial search of the electronic databases identified 945 records. After removing duplicates and screening titles and abstracts, 142 potentially relevant studies were retained. Following full-text assessment based on the predefined inclusion and exclusion criteria, 28 studies were excluded. Ultimately, 12 studies were included in the meta-analysis (15, 18–28). The study selection process is illustrated in Figure 1.

### Study characteristics

Across the 12 included studies, the sample comprised 3,209 participants in the experimental group and 3,174 participants in the control group. The mean age of the enrolled patients ranged from 52.3 to 82.6 years, and the proportion of male participants varied between 48.1% and 70.0%.

### Results of bias assessment

**Figure 2:**
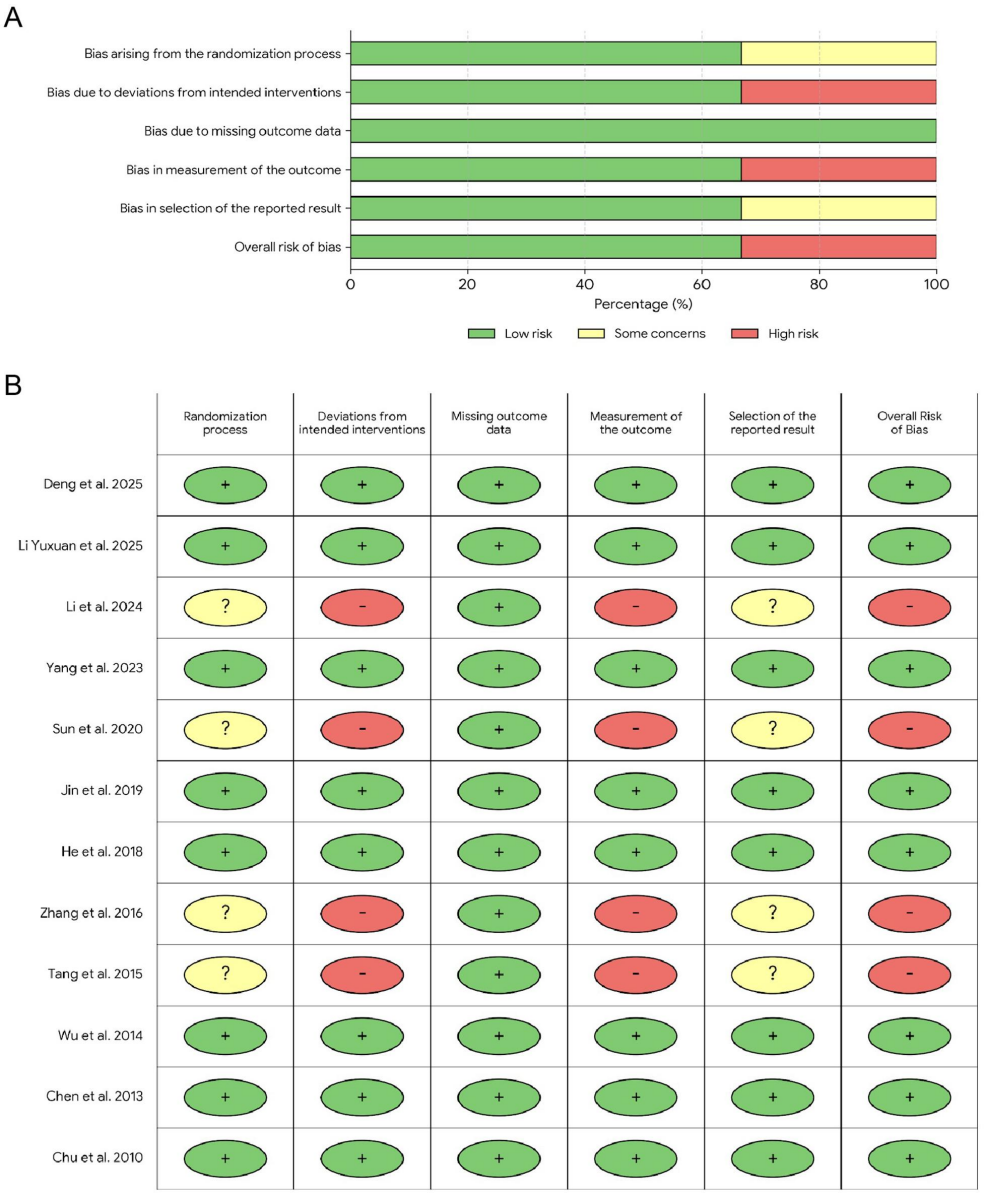
Risk of bias assessment. **(A)** Risk of bias graph: review authors' judgements about each risk of bias item presented as percentages across all included studies. **(B)** Risk of bias summary: review authors' judgements about each risk of bias item for each included study.

The methodological quality of the included RCTs was assessed using the RoB 2. Overall, one study was rated as low risk of bias, four studies were rated as moderate risk, and two studies were rated as high risk. The most common issues included the lack of blinding and inadequate allocation concealment, which are known to introduce biases in RCTs. No evidence of funding bias, incomplete outcome data, or early termination bias was found. Given these findings, the overall risk of bias was considered moderate, and no study was excluded based on quality concerns. A summary of the potential sources of bias and their corresponding proportions is presented in Figure 2.

### Results of the meta-analysis

**Figure 3:**
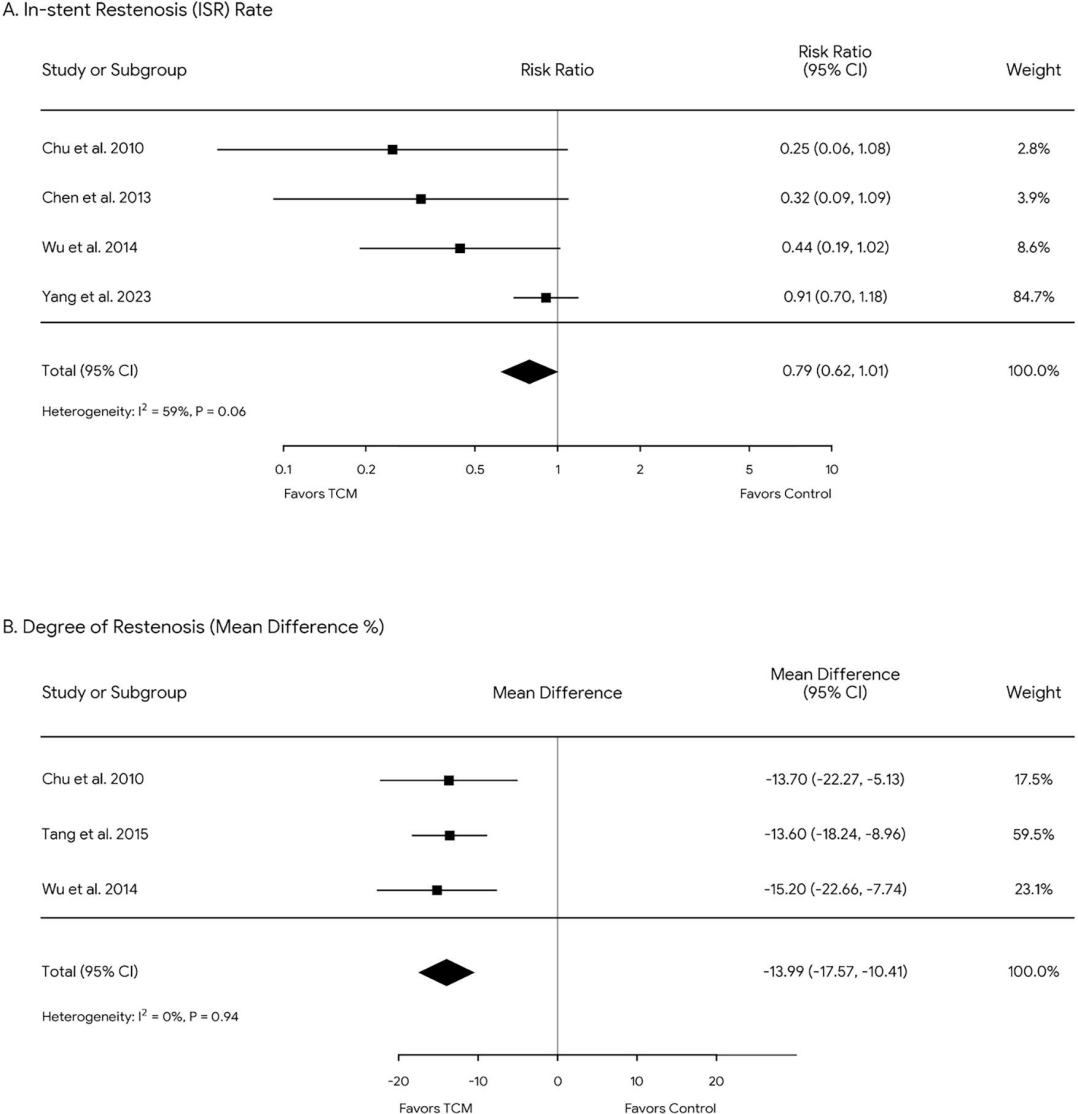
Forest plots of ISR rate **(A)** and restenosis degree **(B)** across all included studies. Diamonds in the central vertical lines represent pooled risk ratios (RR) and mean differences (MD) with corresponding 95% confidence intervals.

The incidence of ISR was significantly lower in the experimental group than in the control group. Furthermore, the experimental group showed a statistically significant reduction in the degree of restenosis compared with the control group (Figure 3).

### Major adverse cardiovascular events

**Figure 4:**
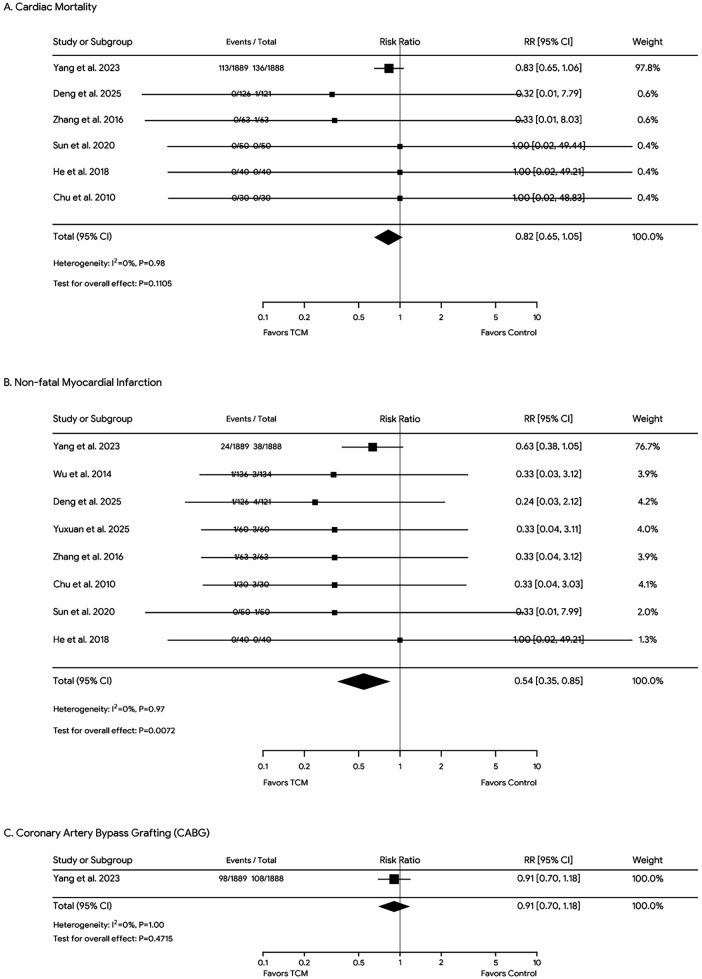
Forest plots of the mortality **(A)**, non-fatal MI rates **(B)**, and Coronary Artery Bypass Grafting (CABG) rates **(C)** across all included studies. Diamonds in the central vertical lines represent pooled risk ratios (RR) with corresponding 95% confidence intervals.

Statistical analysis showed no significant difference in mortality (*P* > 0.05), while the incidence of non-fatal myocardial infarction was significantly lower in the TCM group compared with the control group (*P* < 0.05). However, patients who received TCM treatment showed no significant differences (*P* > 0.05) in the incidence of CABG compared with the control group (Figure 4).

### Publication bias

**Figure 5:**
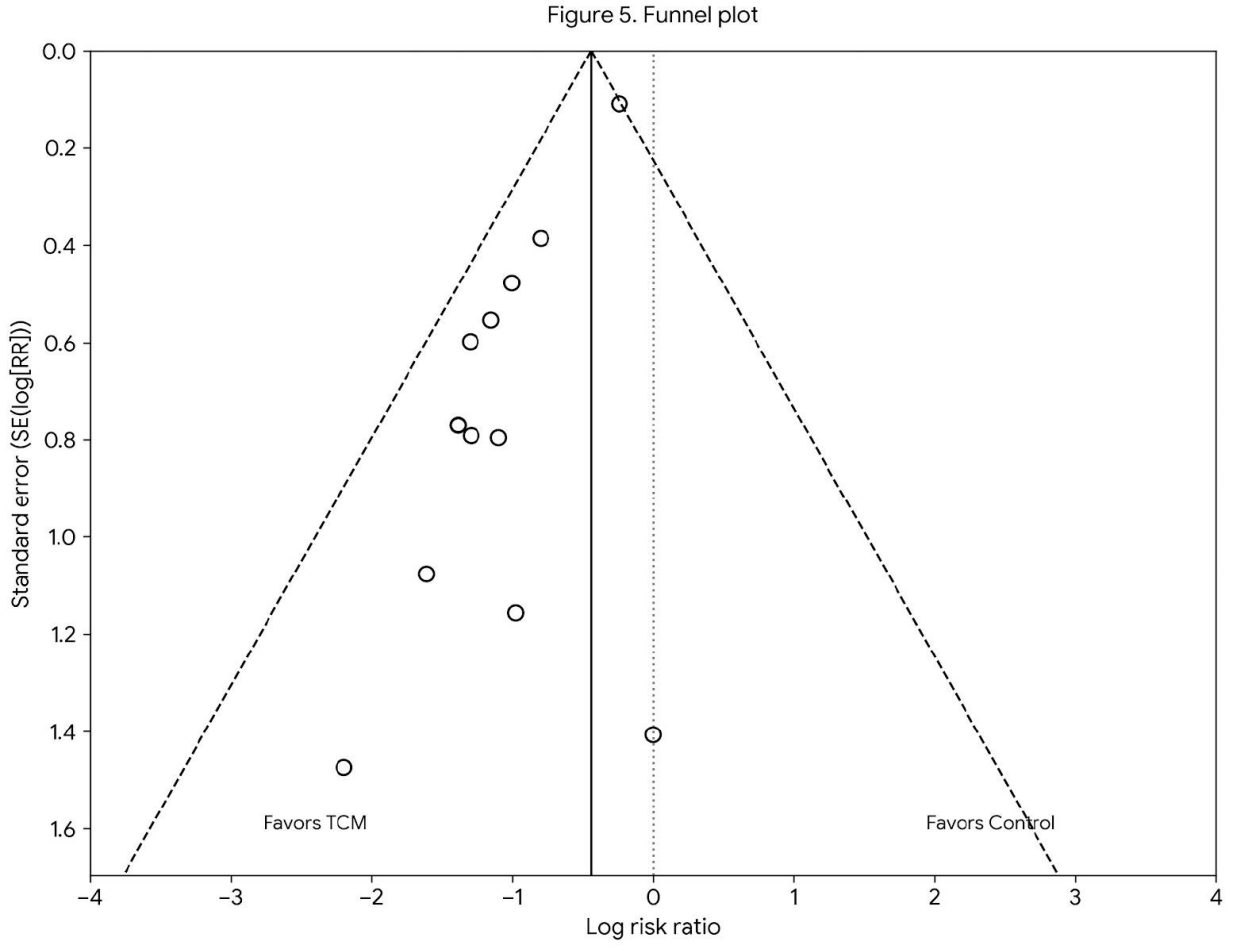
Funnel plot for publication bias in all included studies.

The funnel plots generated from the included studies appeared symmetrical, indicating that the studies were evenly distributed around the pooled effect size and suggesting a minimal risk of publication bias. No significant publication bias was observed upon visual inspection of the funnel plots (Figure 5). In the absence of substantial publication bias, the findings of the meta-analysis are more likely to be reliable and robust, thereby providing stronger support for the research question under investigation.

### Addressing high heterogeneity

To assess heterogeneity across the included studies, we calculated the I² statistic, which evaluates the proportion of total variation due to heterogeneity rather than chance. Although some degree of heterogeneity was observed, with an *I*^2^ value suggesting moderate variability across studies, we chose to use a random-effects model to account for this variability. This approach is commonly used in meta-analyses when studies are expected to be heterogeneous due to differences in study populations, interventions, or methodologies. Additionally, to further explore the impact of heterogeneity, we performed a visual inspection of the funnel plots and conducted Egger's test for publication bias. No significant publication bias was detected, supporting the robustness of the pooled results despite the observed heterogeneity.

## Discussion

CHD in modern medicine corresponds to the TCM concepts of chest Bi and angina. From a modern pathophysiological perspective, this correlation reflects the shared clinical features of myocardial ischemia and coronary circulation disorders (29). In Jin Gui Yao Lue, Zhang Zhongjing described pulse manifestations consistent with CHD as early as the Han Dynasty. According to TCM theory, the pattern of “Yang deficiency with Yin constraint” can lead to chest pain and discomfort due to insufficient Yang in the chest and stagnation of pathogenic Yin. This condition, characterized as Yang deficiency of the jiao, reflects an imbalance caused by the accumulation of cold in the lower body and inadequate Yang Qi in the upper body (30). Biologically, “Yang deficiency” aligns with impaired myocardial bioenergetics and hypoperfusion, while “Yin constraint” corresponds to the retention of pathological metabolic products. Professor Zhang Minzhou has noted that the pathogenesis of ISR involves multiple TCM patterns beyond “Qi deficiency and blood stasis,” such as “Qi deficiency,” “retained fluid,” and “phlegm obstruction.” (31) Mild Yang deficiency, Yin deficiency, and phlegm accumulation are believed to contribute to blood stasis. Similarly, Professor Tie-Tao Deng's research suggests that both deficiency and excess pathogenic factors participate in ISR development, with PCI serving as a direct triggering factor that disrupts vascular patency (32). This mechanical disruption aggravates the local inflammatory response and endothelial injury, which are recognized in Western medicine as key drivers of restenosis. Despite advances in interventional therapy, Li Shilin et al. (33). reported that blood stasis and phlegm turbidity remain the predominant pathological consequences. In the context of cardiovascular pathology, “Blood stasis” is closely associated with platelet activation, hypercoagulability, and microthrombosis, whereas “phlegm turbidity” reflects lipid metabolic disorders and the accumulation of inflammatory cytokines.

This TCM-based approach aims to reduce ISR and stabilize coronary artery disease. According to TCM theory, deficiency and excess share a common etiology. A clinical study involving 107 patients found that ISR after PCI is associated with deficiencies of Qi, Yin, and blood, and involves dysfunction of the lung, spleen, kidney, and liver systems. In this context, “deficiency” refers to insufficiency of Qi, blood, Yin, and Yang in the Zang-fu organs, whereas “excess” denotes Qi stagnation, cold coagulation, blood stasis, and water retention. These deficiencies contribute to the development of ISR in CHD patients following PCI (34). Similarly, Wang Shihan et al. reported that the post-PCI pathogenesis of CHD is characterized by both pathogenic factors and deficiency, as well as primary and secondary deficiencies, which align with the TCM theory of Yin–Yang imbalance (35). Blood stasis, Qi and blood deficiency, Yin–Yang disharmony, excessive fluid retention, phlegm turbidity, and heat toxin constitute the major syndromic components. Qi deficiency is considered the fundamental cause of CHD after PCI, leading to inadequate Qi to promote blood circulation and resulting in stagnation of blood flow. This stagnation exacerbates endothelial dysfunction and promotes the formation of neointimal hyperplasia. In the early stage, blood stasis is the predominant pathological factor. Consistent with these findings, Li Shilin observed that CHD patients before and after PCI predominantly exhibit Qi deficiency syndromes, with clinical manifestations arising from insufficiency of Zheng Qi and imbalance of Yin and Yang.

PCI can effectively remove obstructions in adjacent coronary arteries, improve blood flow, preserve myocardial tissue, and alleviate clinical symptoms such as chest tightness and chest pain. In TCM theory, similar therapeutic principles are reflected in treatments aimed at resolving blood stasis, dispersing clots, promoting blood circulation, and eliminating pathogenic factors. These TCM therapeutic strategies do not merely equate to mechanical clearance but exert their effects by regulating molecular pathways. For instance, “resolving blood stasis” has been pharmacologically linked to the inhibition of platelet aggregation and the downregulation of inflammatory cytokines, which are critical in the early phase of vessel injury. However, PCI targets only the focal diseased segments of the coronary arteries and cannot halt the progression of atherosclerosis throughout the entire coronary system. In addition, stent implantation may induce inflammatory reactions that contribute to the progression of atherosclerotic plaque. Procedural endothelial damage and impaired postoperative reperfusion may also occur. While PCI addresses the anatomical stenosis, it cannot reverse the systemic “internal environment” dysfunction described in TCM. The TCM intervention targets this gap by promoting endothelial repair and improving microcirculation, thereby mitigating the “secondary vascular injury” caused by the procedure. As a result, myocardial ischemia—manifesting as angina pectoris, chest tightness, or ISR-related myocardial infarction—can still develop after PCI and may necessitate repeat coronary revascularization (15). Understanding the mechanisms underlying ISR after PCI is essential for improving the prognosis of patients with CHD. Previous studies have suggested that endovascular hyperplasia, influenced by stent material, procedural techniques, and patient-specific factors, is a key contributor to ISR. Post-PCI management of ISR commonly includes anticoagulant, antiplatelet, and lipid-lowering therapies, as well as interventional procedures such as balloon angioplasty, coronary plaque ablation, and repeat stenting (36). Although repeat stenting is often more effective, it may lead to further vascular intimal injury and a higher risk of tertiary restenosis. TCM has been shown to improve quality of life, reduce long-term adverse cardiovascular events, and alleviate ISR-related symptoms following PCI (37). TCM components have been shown to inhibit vascular smooth muscle cell (VSMC) migration and proliferation—a key cellular process in intimal hyperplasia—thus offering a specific countermeasure to the limitations of repeat stenting. When combined with Western medical therapy, TCM may reduce medication dosage requirements, decrease adverse reactions and side effects, and potentially lower treatment costs for patients (38).

The therapeutic effects of Salvia miltiorrhiza are attributed to its key bioactive compounds, primarily Tanshinone IIA and Salvianolic acid B. Pharmacologically, Tanshinone IIA induces vasodilation by inhibiting calcium influx, a mechanism complementary to calcium channel blockers. Simultaneously, Salvianolic acid B exerts antiplatelet activity by suppressing P-selectin expression. Unlike conventional antiplatelet agents that target specific receptors, these compounds offer a multi-target approach that may synergistically reduce thrombosis without significantly increasing bleeding risk (39). Compound preparations containing Salvia miltiorrhiza, such as Salvia dripping pills, have been shown to improve hemorheology in CHD patients after PCI. Astragalus supports nitric oxide production, mimicking the endothelial-protective effects of ACE inhibitors but with a milder hemodynamic profile. Crucially, it demonstrates a synergistic interaction when combined with standard post-PCI regimens, potentially enhancing plaque stabilization without inducing the adverse hypotension or bradycardia often seen with aggressive Western pharmacotherapy (40). In this study, a combination of Western medicine and TCM was used to manage cardiovascular complications following PCI. Compared with Western medicine alone, TCM combined with Western medicine significantly reduced ISR and non-fatal myocardial infarction (MI) after PCI (*P* < 0.05). However, no significant differences were observed between the two groups regarding cardiogenic mortality or CABG (*P* > 0.05). While severe three-vessel disease or inadequate reperfusion may lead to persistent adverse outcomes regardless of intervention, TCM serves as a complementary therapy beneficial in both the acute and recovery phases. Short-term reductions in ISR and non-fatal myocardial infarction after PCI may contribute to decreased cardiac mortality, although long-term benefits remain unclear. Therefore, hospitalized CHD patients may benefit from combined TCM and Western medical therapy. This meta-analysis demonstrates that integrating Western medicine with TCM can reduce ISR and non-fatal myocardial infarction after PCI. Notably, no serious adverse events were reported in the included studies regarding the use of these TCM interventions.

There was no statistically significant difference in outcomes with traditional Western medical treatment alone. From a health economic perspective, although adding TCM involves an initial cost, it may improve overall cost-effectiveness by reducing the rate of ISR and the subsequent need for expensive repeat revascularization (Target Lesion Revascularization). Furthermore, integrated therapy aligns with patient preferences, particularly among populations who value holistic care and may have poor tolerance for high-intensity pharmacotherapy due to side effects. Nevertheless, widespread practical implementation faces challenges, primarily regarding the standardization of herbal preparations and quality control across different clinical settings. Therefore, hospitalized CHD patients may benefit from combined TCM and Western medical therapy, provided that high-quality, standardized TCM formulations are utilized. This meta-analysis demonstrates that integrating Western medicine with TCM can reduce ISR and non-fatal myocardial infarction after PCI. These outcomes have the potential to improve both short-term and long-term patient prognosis. Notably, no adverse events have been reported clinically with the use of these TCM interventions.

This study has several limitations that should be acknowledged. First, due to the small number of included studies (12 studies), we were unable to perform subgroup analysis or sensitivity analysis, both of which typically require a larger sample size or more variability across studies. The limited number of studies also restricted our ability to explore potential differences in treatment effects across various patient subgroups or intervention protocols. Second, the available clinical indicators in the included studies were limited. For instance, TCM syndrome scores and direct indicators of cardiac function, such as LVEF and NT-proBNP, were not reported in the included studies. These indicators are essential for evaluating the full efficacy of TCM in post-PCI patients, and their absence represents a significant limitation in our analysis. The lack of these comprehensive clinical measures may have restricted our ability to fully assess the impact of TCM 27on cardiac function and outcomes. Despite these limitations, the findings of this study provide valuable insights into the potential benefits of combining TCM with conventional Western medicine for CHD patients after PCI. Future studies with larger sample sizes, more comprehensive clinical data, and inclusion of relevant indicators are needed to further validate our findings.

## Data Availability

The datasets presented in this study can be found in online repositories. The names of the repository/repositories and accession number(s) can be found in the article/Supplementary Material.
