# Supplementary material for: The effects of Traditional Chinese Medicine on cardiac function after percutaneous coronary intervention: a meta-analysis and systematic review
Source: Front Cardiovasc Med. 2026 Feb 18;13:1619928. doi: 10.3389/fcvm.2026.1619928 (PMC12957210; doi:10.3389/fcvm.2026.1619928)
Supplement: Supplementary file 1 [file Table1.docx]

**Supplementary Table S1. Detailed Search Strategies for Each Database**

| **Database** | **Search Query / Strategy** | **Filters / Limits** |
| --- | --- | --- |
| **PubMed** | ((("Coronary Disease"[MeSH Terms] OR "Coronary Heart Disease"[Title/Abstract] OR "CHD"[Title/Abstract] OR "Myocardial Ischemia"[MeSH Terms]) AND ("Percutaneous Coronary Intervention"[MeSH Terms] OR "PCI"[Title/Abstract] OR "Stents"[MeSH Terms] OR "Angioplasty"[Title/Abstract])) AND ("Medicine, Chinese Traditional"[MeSH Terms] OR "Drugs, Chinese Herbal"[MeSH Terms] OR "Traditional Chinese Medicine"[Title/Abstract] OR "Chinese Herbal Medicine"[Title/Abstract] OR "TCM"[Title/Abstract])) AND ("Randomized Controlled Trial"[Publication Type] OR "Randomized"[Title/Abstract] OR "Placebo"[Title/Abstract]) | Publication Date: Inception to March 31, 2025; Language: English |
| **Embase** | ('coronary heart disease'/exp OR 'coronary artery disease':ab,ti OR 'percutaneous coronary intervention'/exp OR 'stent'/exp) AND ('traditional chinese medicine'/exp OR 'chinese herbal medicine'/exp OR 'herbal medicine':ab,ti) AND ('randomized controlled trial'/exp OR 'randomization'/exp) | Publication Date: Inception to March 31, 2025; Language: English |
| **Cochrane Library** | ([mh "Coronary Disease"] OR "CHD":ti,ab) AND ([mh "Percutaneous Coronary Intervention"] OR "PCI":ti,ab) AND ([mh "Medicine, Chinese Traditional"] OR "Chinese Medicine":ti,ab) in Trials | Publication Date: Inception to March 31, 2025 |
| **CNKI (China National Knowledge Infrastructure)** | (SU='冠心病' OR SU='缺血性心脏病') AND (SU='经皮冠状动脉介入' OR SU='PCI' OR SU='支架') AND (SU='中医药' OR SU='中西医结合' OR SU='中药' OR SU='汤剂') AND (SU='随机' OR SU='对照') | Publication Date: Inception to March 31, 2025; Language: Chinese |
| **Wanfang Data** | (Subject:"冠心病" OR Title:"CHD") AND (Subject:"PCI" OR Subject:"介入治疗") AND (Subject:"中医药" OR Subject:"中西医结合") AND (Subject:"随机对照") | Publication Date: Inception to March 31, 2025; Language: Chinese |
| **ClinicalTrials.gov** | Condition: "Coronary Heart Disease" AND Intervention: "Traditional Chinese Medicine" OR "Herbal Medicine" | Status: Completed; Study Type: Interventional (Clinical Trial) |
